# Supplementary material for: Factors affecting the prevention of unwanted pregnancies among young adolescents in secondary schools in the Eastern Province of Rwanda: An explorative qualitative study
Source: PLoS One. 2024 Jul 26;19(7):e0301248. doi: 10.1371/journal.pone.0301248 (PMC11280217; doi:10.1371/journal.pone.0301248)
Supplement: S1 File — (DOCX) [file pone.0301248.s001.docx]

**English version: Semi- structured Interview guide**

**Part 1: Socio-demography information**

| 1.1 Research participant number: | 1.6 Interviewer’s name: |
| --- | --- |
| 1.2 Date: | 1.7 Age: |
| 1.3 School name: | 1.8 Sex: |
| 1.4 Start Interview: | 1.9 Class for participant: |
| 1.5 End Interview: | 1. 10 Religion: |

PART 2. Inf**ormation about sexual and reproductive health changes during adolescence**

1. As adolescent boys and girls transition into young adulthood, what types of information are essential to assist them in navigating this period of change, specifically in relation to managing the physical and psychological transformations that occur during adolescence?
2. Can you tell me some of the strategies that you can use to prevent sexually transmitted infections and unwanted pregnancies among young girls?
3. What are some of the obstacles that adolescent girls and boys encounter during their teenage years that could impact their sexual and reproductive well-being?
4. Which of the obstacles you have identified do you believe adolescent girls and boys may lack awareness of in terms of the impact they can have on their sexual and reproductive health as they move from puberty to late adolescence?

**Thank you for the role you have played in providing the information pertaining to sexual and reproductive health changes among young girls/boys who have reached adolescent period**.
