# Supplementary material for: Factors affecting the prevention of unwanted pregnancies among young adolescents in secondary schools in the Eastern Province of Rwanda: An explorative qualitative study
Source: PLoS One. 2024 Jul 26;19(7):e0301248. doi: 10.1371/journal.pone.0301248 (PMC11280217; doi:10.1371/journal.pone.0301248)
Supplement: Attachment — Submitted filename: Rebutal letter.docx [file pone.0301248.s002.docx]

**Point-by-point response to reviewers’ comments**

| **Comments** | **How the comments have been addressed** |
| --- | --- |
| **Academic Editor** | |
| If you would like to make changes to your financial disclosure, please include your updated statement in your cover letter. Guidelines for resubmitting your figure files are available below the reviewer comments at the end of this letter. | *We did not change our financial disclosure because the way it is stated reflected our situation.* |
| If applicable, we recommend that you deposit your laboratory protocols in [protocols.io](http://protocols.io/) to enhance the reproducibility of your results. Protocols.io assigns your protocol its own identifier (DOI) so that it can be cited independently in the future. For instructions see: <https://journals.plos.org/plosone/s/submission-guidelines#loc-laboratory-protocols>. Additionally, PLOS ONE offers an option for publishing peer-reviewed Lab Protocol articles, which describe protocols hosted on [protocols.io](http://protocols.io/). Read more information on sharing protocols at <https://plos.org/protocols?utm_medium=editorial-email&utm_source=authorletters&utm_campaign=protocols>. | *Not applicable as our study was qualitative.* |
| When submitting your revision, we need you to address these additional requirements.  1. Please ensure that your manuscript meets PLOS ONE's style requirements, including those for file naming. The PLOS ONE style templates can be found at  <https://journals.plos.org/plosone/s/file?id=wjVg/PLOSOne_formatting_sample_main_body.pdf> and  <https://journals.plos.org/plosone/s/file?id=ba62/PLOSOne_formatting_sample_title_authors_affiliations.pdf>  2. Please include captions for your Supporting Information files at the end of your manuscript, and update any in-text citations to match accordingly. Please see our Supporting Information guidelines for more information: <http://journals.plos.org/plosone/s/supporting-information>. | *Thank you for raising this important issue. In the revised manuscript we followed the journal’s style requirements throughout. In addition, the caption of the supporting information files were renamed according to the Journal’s requirements.* |
| ***Reviewer 1*** | |
| As stated, this article presents part of the results of a more comprehensive study. Maybe this issue has caused ambiguity and some questions: - What exactly did the research team mean from the “perspectives of young adolescents”? - How was the interview and managing the meetings guide compiled and what questions did it include? - Certainly, significant data was obtained from the 12 focus group discussion sessions. What are the special features of the three presented “themes” that are reported separately from other research findings? Although the title of the third theme is also very general and non-specific. | *Thank you for raising this important issue regarding the fact that this study presents part of the results of a larger study. We indicated in the methodology that:*  *‘*The initial phase of the project centered on gathering young adolescents’ understanding of the physiological and emotional changes taking place during different stages of adolescence. Additionally, it explored adolescents’ personal experiences with these changes and examined the strategies they employed to navigate through them. The second phase which is reported in the current pater focused on factors affecting young adolescents’ prevention of unwanted pregnancies and sexually transmitted infections including HIV/AIDS and what they need to maintain a healthy sexual and reproductive health.’ (see page 7)  *In order to match our study with the results and the discussions thereof, we changed the title for this manuscript to:*  *‘***Factors affecting the prevention of unwanted pregnancies among young adolescents in secondary schools in the Eastern Province of Rwanda: An explorative qualitative study’**  We have also restructured the reporting of the results. In the current revised manuscript version, theme 1 has been subdivided into two sub-themes, theme 2 into three sub-themes, and theme 3 with no sub-themes. This was done after adding more results to respond to the concerns raised by the reviewer. (For details, see results chapter from pages 13-21) |
| - It seems that the researchers' main focus was on unwanted pregnancy (also its outcomes) and HIV/AIDS. Based on this, it was concluded that the awareness of adolescents was acceptable. But with the "comprehensive sexuality education" perspective, we know that having awareness alone is not enough to protect teenagers. Are concepts such as life skills, sexual and reproductive health rights, responsibility, assertiveness and saying no, etc. addressed in this study? | *Thank you for this important commentary. In the limitations of this study, we acknowledged that our study did not cover concepts like life skills, sexual and reproductive health rights, and assertiveness and we recommended that further research is needed to investigate these concepts in the context adolescents’ sexual and reproductive health education. (see page 24 for details)* |
| ***Reviewer 2*** | |
| There are a few things that would strengthen this manuscript, including being careful about terminology, specific about approach, and including some more context in the conclusion about what programs have (or have not been successful) and their target audience. | *Thank you for the constructive comment. In the revised version, the raised issues were taken into consideration.* |
| Abstract: “and 4% are currently pregnant with their first child” | *Addressed. See page 2* |
| - This conclusion in the first paragraph feels a bit abrupt, consider removing “thus” and making it the first sentence of the next paragraph “Thus, in order to promote the well-being of adolescents, it is essential to safeguard their sexual and reproductive health.” | *Addressed. See page 4* |
| - Rather than “Research have also revealed that” should be “Research has also revealed that” | *Addressed. See page 5* |
| - This reads as though >6 million pregnancies resulted in unsafe abortions … “These unintended pregnancies resulted in 12 million births, and 55% of them ended in unsafe abortions[9].” | *Addressed. See page 5* |
| - This sentence is confusing, were approx. half unintended or approx half from sub-saharan Africa? “In 2019 alone, it was estimated that 21 million pregnancies occurred in this age group, with approximately half of them being unintended were from Sub-Saharan Africa[10].”  - Consider defining unintended pregnancies in the intro – specifically these can be unwanted, or can be unplanned (which are qualitatively different). Not sure if that distinction will be made in the qualitative data but unplanned is not necessarily unwanted. | *The ambiguation in this sentence has been addressed by removing the phrase ‘these unintended pregnancies resulted in 12 millions births’ (see page 5)* |
| - Also may be useful to define sexual and reproductive health – think this is encompassing access to care, access to contraception, prevention of STDs? As I read through the intro, I am starting to think the specific research question is about risk factors for teenage pregnancy, not a the broader concept of sexual and reproductive health. Obviously its helpful to situation one within the other, but it would be helpful to re-frame this so its clear what the article addresses, and why that is important in the larger picture for adolescents. | *Thank you for underlining the need to define the terms ‘unintended pregnancies, and sexual and reproductive health’ in the context of this study. We provided that definition on page 4* |
| - For inclusion criteria “capable of expressing themselves” I am not sure how that was assessed or defined. Then it states “Within the context of this study, purposive sampling was employed to identify and select adolescent boys and girls who were capable of articulating their viewpoints and recounting their personal experiences regarding the issues affecting their sexual and reproductive health within their respective residence areas.” But this makes it sound like only articulate adolescents were included, which makes me concerned about bias … | *Thank you for the advice. We have removed the phrase ‘capable of expressing themselves. See page 8 for details.* |
| - How was sample size determined? | *Thank you for this observation. We have added a section about sample size determination. See page 9 for details.* |
| Discussion/conclusion: - Were gender differences seen in the types (or ways) themes were discussed? | *Thank you. Since our primary objective was to identify factors affecting the prevention of unintended pregnancies and STIs, we did not discuss the gender differences in the themes discussed because we thought that there no major differences from the viewpoints of male and female adolescents.* |
| - Nevertheless, our research did not address the determinants of risky sexual behavior among young people attending secondary schools in the Eastern province of Rwanda. - “consisted of sexually active young individuals” I don’t think this was inclusion criteria? | *Thank you for this observation. The comment was considered and we removed the two sentences that focused on that aspect(see page 23).* |
| - In the conclusion, I question whether a program for parents is the best approach given the findings. You also haven’t cited any programs that have done this successfully (or not), but given the cultural factors and the difficulty of accessing parents for a training, I would propose that providing information and training directly to students in schools may be more effective (and much easier logistically). | *Thank you for your observation. As you highlight it, educational interventions targeting students in schools would be more effective and much easier logistically. We corrected accordingly. (See page 25)* |
