# Supplementary material for: Factors affecting the prevention of unwanted pregnancies among young adolescents in secondary schools in the Eastern Province of Rwanda: An explorative qualitative study
Source: PLoS One. 2024 Jul 26;19(7):e0301248. doi: 10.1371/journal.pone.0301248 (PMC11280217; doi:10.1371/journal.pone.0301248)
Supplement: Attachment — Submitted filename: Rebutal letter to editors-Thierry et al.2024-14-07-2024.docx [file pone.0301248.s003.docx]

**Point-by-point response to reviewers’ comments**

| **Comments** | **How the comments have been addressed** |
| --- | --- |
| **Reviewer 1** | |
| The revised article provides more information and is more coherent. Mentioning details about the questions and topics of focused group discussions could help improve the article. | *Thank you for the feedback. We have included the interview guide used in the supplementary information (see supplementary information 1)* |
| **Reviewer 2** | |
| Thank you for this revised manuscript. Several revisions I think were helpful for clarity. I do have a few additional comments, below.  • This edit: “an unwanted pregnancy is described as a pregnancy that occurs before they have reached physical and mental maturity to fully understand the implications of their actions and provide informed consent.” With current edits, I think this sentence can just be dropped. | *Thank you. The sentence was deleted. (see page 4)* |
| Paragraph 2 of the intro, there are several acronyms used that are not spelled out. | *Thank you for the comment. The abbreviations have been spelled out in page 4.* |
| This section in the intro is still a bit confusing: “In 2019 alone, it was estimated that 21 million pregnancies occurred in this age group, with approximately half of them being unintended were from Sub-Saharan Africa[14].” Were approximately half unintended or half were from SSA? | *Thank you for the observations. Corrections were made to make the statement clearer. (see page 5 for edits made)* |
| Similarly, I am not sure what “These unintended pregnancies resulted in 12 million births, and 55% of them ended in unsafe abortions[13].” Refers to but it implies that half of the global pregnancies were unintended (so not sure what proportion are from SSA), and that of the half that were unintended, half of those ended in unsafe abortions? That seems extremely high to me, so I looked up the reference, and two things 1) Ref 13 looks incomplete in the reference list and 2) the reference said 55% resulted in abortions not that these were unsafe, but that some of these were unsafe. | *Thank you for this observation. The confusion has been address and the reference was corrected. (see page 5) the reference list has been revised to make reference 13 complete.* |
| Some specificity in the intro would be helpful, only half (I think) of pregnancies are unintended, which means some are intended. Those that are intended may still threaten well-being or economic attainment, but terminology used and argument don’t make any distinction. | *Thank you for the observation. With the revised version (with the previous 2 comments), this issue has been addressed.* |
| Consider being very explicit at the end of the intro – its there but could be more clear – what this article was aiming to do by literally saying, “Therefore, the current study aimed to … | *Thank you for this observation. The issue raised has been addressed on page 6 of the current version.* |
| “Subtheme 2: Young adolescents are aware of the negative outcomes associated with poorly managed adolescence” the phrase “poorly managed adolescence” is a bit vague I wonder about rather saying “poorly managed sexuality during adolescence”? | *Thank you for the suggestion. Comment considered. See page 14.* |
| Here “In order to address this vulnerability, male participants underscored the importance of providing comprehensive education on sexuality and reproductive health” you say male participants but almost all quotes are from females (which does make more sense to me) | *Thank you for the observation. We have changed male participants to ‘participants’ as this was a common perspective by both male and female participants. See page 26.* |
| Re-orient the reader to the goal of the paper at the start of the discussion before diving into results. | *Thank you for observation. The comment has been taken into consideration. See page 22* |
| This sentence is a bit long and confusing: “Therefore, basing on this finding, we suggest that interventions targeting the reduction of STI and HIV transmission, along with the prevention of unintended pregnancies among adolescents, should be based on the current awareness that young individuals possess regarding the negative outcomes associated with risky sexual behaviors.” | *Thank you for this comment. We have made edits to make the sentence short and comprehensible. (see page 23)* |
| This is still in the manuscript: - “consisted of sexually active young individuals” I don’t think this was inclusion criteria? | *Thank you for this observation. We have addressed this on page 23.* |
